# Primary Small Cell Carcinoma in Urinary Bladder: A Rare Case

**DOI:** 10.1155/2015/789806

**Published:** 2015-12-16

**Authors:** Ahmet Çamtosun, Huseyin Çelik, Ramazan Altıntaş, Nusret Akpolat

**Affiliations:** ^1^Turgut Özal Medical Center, Department of Urology, Malatya, Turkey; ^2^Turgut Özal Medical Center, Department of Pathology, Malatya, Turkey

## Abstract

Small cell carcinoma of bladder, which does not have a common and accepted treatment protocol, is a rare and highly aggressive tumor. It is mostly pulmonary originated; however, it can rarely be seen in extrapulmonary sites. We presented an interesting and uncommon case, in which the transitional cell tumor was found in the transurethral resection specimen, but the small cell carcinoma was detected in the final radical cystectomy material.

## 1. Introduction

Small cell carcinoma is less than 1% in urinary bladder tumors and is very aggressive and refractory to treatment due to its higher metastatic capability compared to other common bladder tumors [1]. When it is diagnosed, the disease is mostly in the metastatic stage, so the patients generally have a poor prognosis. To improve the cure chance or life expectancy, a multidisciplinary approach including radical cystectomy, chemotherapy, and radiation therapy should be initiated as soon as possible [2, 3].

## 2. Case Report

**Figure 1:**
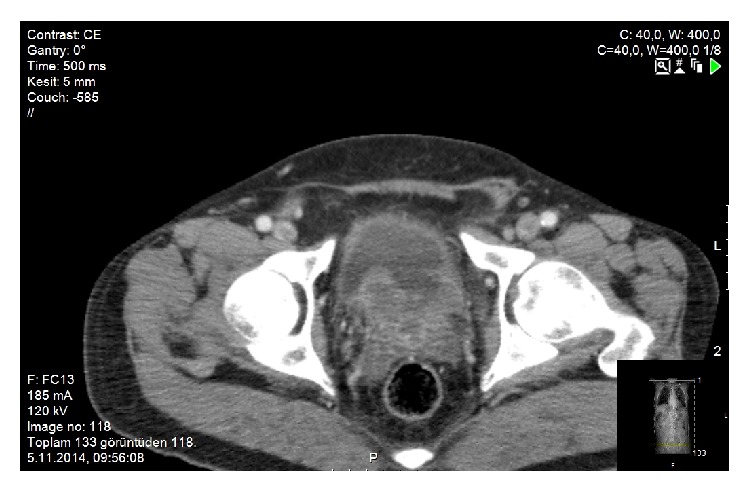
Polypoid mass extending to the lumen of the bladder base.

**Figure 2:**
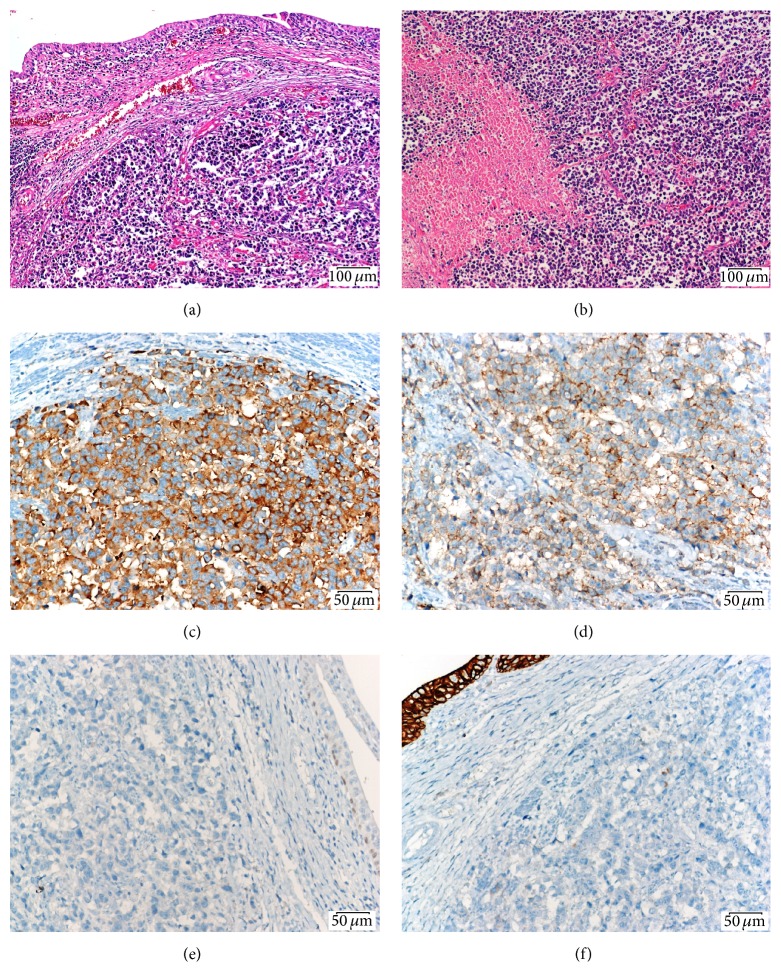
(a) Surface infiltrating urothelial lining laminated propria below shows the small round malignant tumor infiltration (Hematoxylin Eosin stain, ×100). (b) Multiple small round necrotic areas were seen in the malignant tumors (Hematoxylin Eosin stain, ×100). (c) The synaptophisin positivity in neoplastic cells (immunohistochemical stain, ×200). (d) Neoplastic cells in diffuse moderate expression of CD56 membranous (immunohistochemical stain, ×200). (e) This p53 weak expression in basal area is considered negative staining. (immunohistochemical stain, ×200). (f) In general the neoplastic cells negative CK-7 expression (immunohistochemical stain, ×200).

65-year-old male was admitted to our urology department with hematuria. A 4 cm polypoid mass was detected in urinary bladder on computed tomography (CT) (Figure 1) and the patient had cystoscopy and transurethral resection (TUR) of the mass. The pathology of resected mass was high grade urothelial carcinoma (TCC) invading muscularis propria. The patient's whole body scan had no evidence of metastasis. Patient underwent radical cystoprostatectomy and urinary diversion with ileal loop (Wallace ureteroileostomy) and extended lymph node dissection. The duration of radical cystectomy surgery was six hours and there was a negligible bleeding during the operation. In the pathological evaluation, there was primary small cell carcinoma in cystectomy specimen and metastatic invasion in 3/4 of the right obturator and iliac lymph nodes (Figure 2). In the postoperative positron emission tomography (PET) CT taken before the chemotherapy planning, there was small millimetric lung metastases. A chemotherapy including etoposide and cisplatin was started at 14th postoperative day.

## 3. Discussion

Small cell carcinoma of bladder was firstly reported in 1981 by Cremer et al. [4]. There were 600 cases reported till now. This is a very aggressive tumor and generally has a poor prognosis. More than 60% of the reported patients were metastatic at diagnosis [3].

Small cell carcinoma of bladder has similar characteristics of age, sex, and symptoms to TCC. In addition the radiological images of these 2 different tumors are also the same. They can be distinguished by histopathologic examination. Small cell carcinoma of bladder is more rare and aggressive than TCC [5]. Small cell carcinoma of bladder is mostly found together with TCC in a form of a large mass or rarely alone in the histopathologic examination of cystectomy specimen; however, it can be diagnosed accompanied with TCC by TUR of the bladder mass. Even if TCC was detected in the first cystoscopic evaluation, re-TUR should be done to identify the concomitant different tumor like small cell carcinoma and to determine possible muscle invasive TCC. In our case, small cell carcinoma was diagnosed in the pathologic evaluation of the cystectomy specimen.

The pathological diagnosis of this tumor is difficult and some immunohistochemical staining techniques can be required to differentiate these 2 tumors [5]. TMPRSS2-ERG fusion gene is generally used in the diagnosis of small cell carcinoma of prostate rather than bladder [6]. Epidermal growth factor receptor (EGFR) protein expression and gene amplification were evaluated in small cell carcinoma of bladder, and presence of these was correlated with the pathological stage of this tumor [7]. On the other hand DNA methylation can be used as a biomarker in the diagnosis, treatment, and follow-up of small cell carcinoma of bladder [8].

In selected patients with localized lower stage small cell carcinoma, TUR, partial cystectomy or radiotherapy can be done. Radical cystectomy is considered as the best method to completely eliminate the small cell carcinoma of the bladder, but it improves survival only in the localized tumor. Moreover, in addition to radical cystectomy, extended lymph node dissection can increase the survival rate. There is no consensus on the treatment protocol of small cell carcinoma of bladder, so the therapy approach has been done according to the previous case reports and the retrospective studies on small cell lung cancer.

Cheng and colleagues reported that there was no significant difference in survival between the patients groups treated by radical cystectomy with chemotherapy and radical cystectomy with chemoradiotherapy [9]. Conversely, in another study, it was reported that adjuvant chemotherapy alone could increase the survival rate more than adjuvant chemoradiotherapy [10]. Moreover, a study conducted in MD Anderson Cancer Centre showed that neoadjuvant chemotherapy supplied 78% disease-free survival rate, but radical cystectomy alone had 36% [11]. On the other hand, a study involving 10 patients with pT3-T4 N0 conducted by Lohrisch et al. demonstrated that chemoradiotherapy without cystectomy supplied 70% 2-year survival rate and 44% 5-year survival rate [12]. In our case, chemotherapy alone was started 2 weeks after the operation and in 12th month of control after the surgery; the disease was in the remission period.

## 4. Conclusion

Small cell carcinoma of the bladder is considered to be extremely aggressive and there is less known information about its pathogenesis and molecular biology. There are few data on the ideal approach for diagnosis and treatment in this tumor. In such cases, urologists, pathologists, and medical oncologists have a big responsibility. With a multidisciplinary approach, early diagnosis and immediate intervention can supply a better survival and a more comfortable life.
